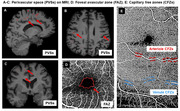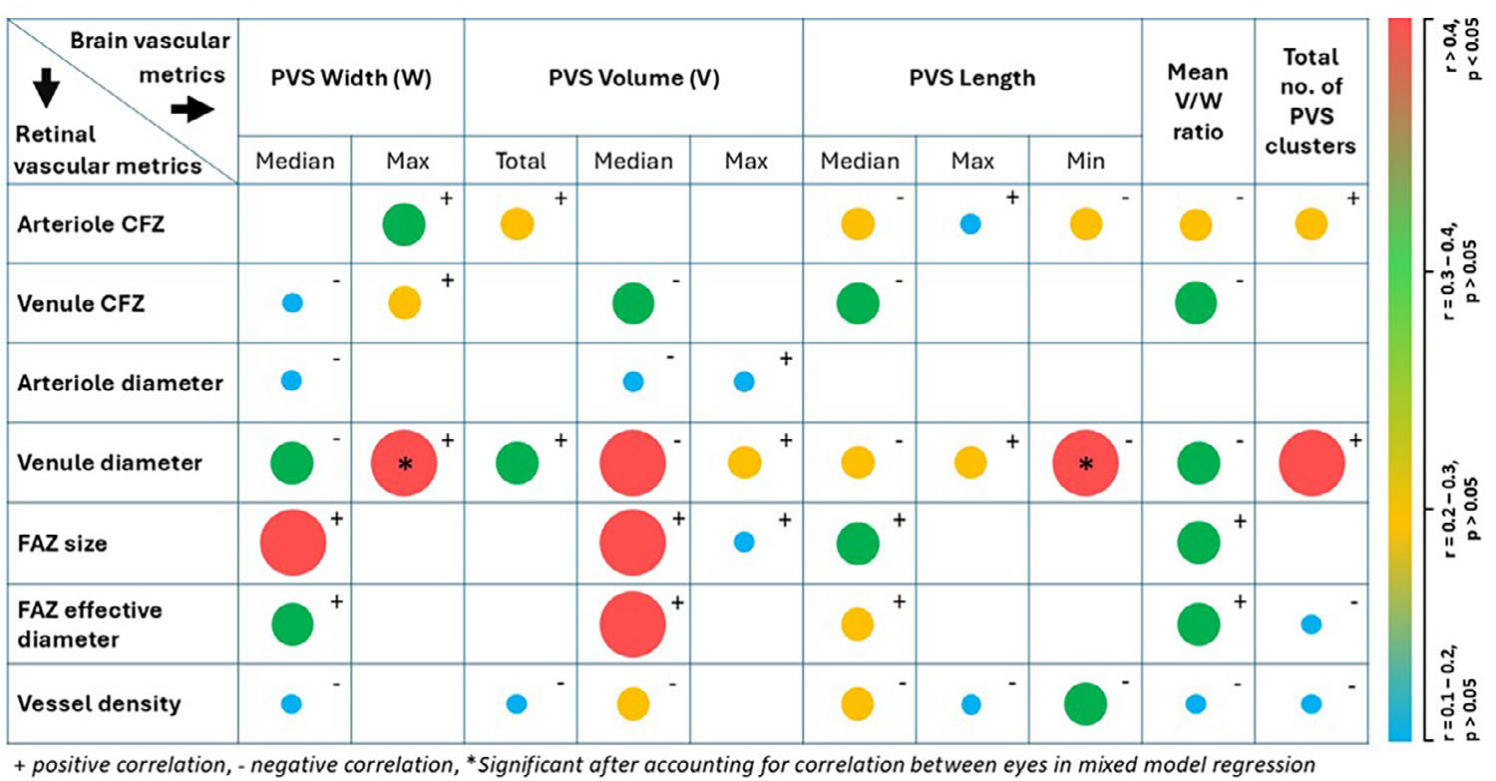# Retinal vascular metrics are associated with enlarged MRI‐visible perivascular spaces in cognitively unimpaired older adults

**DOI:** 10.1002/alz70856_100913

**Published:** 2025-12-24

**Authors:** Edmund Arthur

**Affiliations:** ^1^ University of Alabama at Birmingham, Birmingham, AL, USA

## Abstract

**Background:**

Perivascular spaces (PVSs) in the brain, when enlarged, serve as indicators of microvascular changes associated with aging and neurodegenerative conditions. Given that the retina shares microvascular similarities with the brain, this study aimed to explore the association between retinal vascular metrics, including mid‐peripheral capillary free zones (CFZs) and MRI‐visible PVS burden.

**Method:**

20 × 20 deg optical coherence tomography angiography images of the central fovea and of paired arterioles, venules, and surrounding capillaries inferior to the fovea of 23 eyes of 14 cognitively unimpaired older adults (mean: 64 years) were acquired. Additionally, a 3T GE PET/MR scanner with a 32‐channel head coil was used to obtain brain images. Retinal metrics, including mid‐peripheral CFZs, vessel diameter, and vessel density, were computed using MATLAB, while the foveal avascular zone (FAZ) was quantified with vendor software lasso tool. An established PVS automatic segmentation algorithm provided whole‐brain PVS characterization, including PVS count, volume, length and width. Correlations were assessed using Pearson correlation and mixed model regression.

**Result:**

Although periarteriole CFZ showed a positive trend with PVS width and volume, no significant associations were found. In contrast, perivenule CFZ exhibited a negative trend with median PVS volume and length. Notably, median PVS volume was significantly associated with venule diameter (*r* = ‐0.51, *p* =  0.017), FAZ size (*r* = 0.48, *p* =  0.02), and FAZ effective diameter (*r* = 0.44, *p* =  0.037), while median PVS width was significantly associated with FAZ size (*r* = 0.43, *p* =  0.04). The association between maximum PVS width, minimum PVS length and venule diameter remained significant even after accounting for within‐subject correlation between eyes. Interestingly, age was positively correlated with the total number of perivascular space clusters found in individuals (*r* = 0.54, *p* =  0.008).

**Conclusion:**

Our findings suggest that retinal vascular metrics are significantly associated with MRI‐visible PVS burden in cognitively unimpaired older adults, highlighting the potential of using retinal vascular metrics as surrogate markers for brain vascular changes.